# Foot and ankle pain and risk of incident knee osteoarthritis and knee pain: Data from the Multicentre Osteoarthritis Study

**DOI:** 10.1016/j.ocarto.2021.100210

**Published:** 2021-12

**Authors:** Thomas A. Perry, Neil A. Segal, Catherine Bowen, Lucy Gates, Nigel Arden, Michael C. Nevitt

**Affiliations:** a Nuffield Department of Orthopaedics, Rheumatology and Musculoskeletal Sciences, Botnar Research Centre, University of Oxford, Old Road, Oxford, OX3 7LD, United Kingdom; b Centre for Sport, Exercise and Osteoarthritis Versus Arthritis, Nuffield Department of Orthopaedics, Rheumatology and Musculoskeletal Sciences, University of Oxford, Oxford, United Kingdom; c University of Kansas Medical Centre, Kansas City, KS, USA; d The University of Iowa, Iowa City, USA; e School of Health Sciences, Faculty of Environmental and Life Sciences, University of Southampton, Southampton, United Kingdom; f Centre for Sport, Exercise and Osteoarthritis Versus Arthritis, University of Southampton, Southampton, United Kingdom; g MRC Lifecourse Epidemiology Unit, Southampton General Hospital, University of Southampton, Southampton, United Kingdom; h Department of Epidemiology and Biostatistics, University of California San Francisco, 550 16th St, San Francisco, CA 94158, USA

**Keywords:** Ankle, Pain, Incident, Knee osteoarthritis (OA), Outcomes

## Abstract

**Objectives:**

To examine whether foot and/or ankle pain increases the risk of knee OA.

**Design:**

We utilised longitudinal data from the Multicentre Osteoarthritis Study (MOST); a community-based cohort of risk factors for knee OA. Participants without frequent knee pain (clinic visit only) and radiographic knee OA (RKOA) at baseline and, with no evidence of inflammatory musculoskeletal disease and a history of knee-related surgery were followed for up to 84-months for incident outcomes; i) RKOA (Kellgren-Lawrence (KL) ≥2), ii) symptomatic RKOA (RKOA and frequent pain in the same knee) and iii) frequent knee pain only. At baseline, ankle and foot symptoms were assessed, with knee radiographs and symptoms also assessed at 30, 60 and 84-months. Our exposures included baseline ankle, foot, and ankle and foot pain (participant-level). Associations between foot and/or ankle pain and incident outcomes were assessed using multiple logistic regression, with adjustment for participant characteristics and ankle/foot pain.

**Results:**

No statistically significant associations were observed between ankle, foot and, ankle and foot pain and incident RKOA, respectively. Ankle pain with (2.30, 95% CI 1.13 to 4.66) and without foot pain (OR: 2.53, 95% CI 1.34 to 4.80) were associated with increased odds of incident symptomatic RKOA and frequent knee pain. No statistically significant associations were observed between foot pain and these outcomes.

**Conclusions:**

Ankle pain should be a focus point, more so than foot pain, in the management of knee OA. Future studies should include additional ankle joint-specific symptom questions to better elucidate the knee OA biomechanical pathway.

## 1 Introduction

Osteoarthritis (OA) is the most common musculoskeletal disorder [1], is a global health concern and is a leading cause of joint symptoms and the loss of quality of life [2]. Radiographic knee OA (RKOA), with or without knee joint symptoms, typically progresses and worsens over time and this can ultimately lead to costly knee joint replacement surgery. Understanding risk factors associated with the onset of structural knee OA, with or without symptoms, is a major research focus as it would inform the development of preventative interventions.

In OA, isolated joint pain is uncommon [3,4] and compared to those who have few sites affected, persons reporting multiple sites with joint pain typically have worse function [5], quality of life [6] and worse outcomes following knee replacement [7]. Although it has been established that symptoms and/or structural OA in lower extremity joints (e.g., ankle) can affect other kinematically involved joints of the lower extremities (e.g., knee), most studies have focused on the interaction between the knee and hip; with little attention on the ankle joint.

There is growing evidence to suggest that changes in biomechanics at the ankle are associated with knee OA [[8], [9], [10]]. Kraus et al. reported that pain commonly developed in the ankle in patients with evidence of knee OA [11]. More so, using cross-sectional data from the Osteoarthritis Initiative (OAI), Paterson et al. reported that patients with symptomatic RKOA and foot and/or ankle symptoms had significantly worse general and knee-specific health outcomes compared to patients with just symptomatic RKOA [12]. Again, using data from the OAI, foot and/or ankle pain has been linked with the development of symptomatic RKOA and knee pain in the short-term (4-years) [13], and worsening knee pain in patients with symptomatic RKOA [14]. There is, however, a need to examine the independent effects of ankle and foot pain on knee OA outcomes, including RKOA, using additional study populations and, using long term follow-up data.

In the case of multi-site joint pain, the development of pain at an adjacent site is likely due to patient-specific modifications in loading to avoid pain, which may contribute to structural osteoarthritic changes. This can be seen in cases on unilateral hip OA in which the contralateral hip and knee are at higher risk of developing OA [15,16]. It has been hypothesised that adaptations in gait and loading to avoid joint pain, particularly of the lower-limbs, are person-specific [17] making the identification of patterns of disease development difficult [4]. Thus, there is a need to examine the relationship between ankle and foot pain and knee OA outcomes in multiple study populations. Whilst it is possible that multi-site pain may occur in response to structural osteoarthritic changes, it is also possible that multiple sites with joint pain may result from changes in central pain processing leading to increased pain sensitization [18].

Our aims were to examine the effect of ankle, foot and, ankle and foot pain on incident knee OA outcomes in older adults at elevated risk of knee OA.

## 2 Methods

### 2.1 Study sample

We used longitudinal data from the Multicentre Osteoarthritis Study (MOST); a prospective, observational study of risk factors for knee OA (http://most.ucsf.edu/). Details of the MOST study have been published in detail [19]. In brief, MOST enrolled 3026 community-dwelling adults aged between 50 and 79 years from 2 communities across the United States. At baseline, study participants either had evidence of knee OA or had known risk factors for knee OA and were followed for up to 84-months.

We included participants who were free of whole-knee RKOA and frequent knee symptoms (see next section for definitions) in both knees at baseline. We examined the presence of baseline RKOA at participant-level, and subsequently excluded participants rather than knees as there is evidence to suggest that unilateral knee OA can increase the risk of the developing OA in the contralateral knee [20,21]. Similarly, there is evidence to suggest that unilateral knee pain is associated with incident joint pain on the contralateral side in participants at risk of knee OA [4]. Demographic, clinical, and radiographic characteristics for all participants were captured at baseline and at 15-, 30-, 60- and 84-month visits. We used data at baseline, 30-, 60- and 84-month follow-up as this contained the most complete knee radiographic dataset.

### 2.2 Assessments

#### 2.2.1 Knee radiographs

At baseline and at follow-up, all participants underwent weight-bearing, fixed-flexion posteroanterior and lateral radiographs of the knees. Knee radiographs were graded on a 0–4 scale, across the whole knee joint (including patellofemoral and tibiofemoral regions), using Kellgren & Lawrence (KL) criteria [22]. Radiographic OA of the whole knee was defined as a KL score of ≥2 (in either, or both, the tibiofemoral and patellofemoral joints).

### 2.3 Knee symptoms

Knee symptoms were assessed at each clinic visit using a modified version the National Health and Nutrition Examination Survey (NHANES) questions [23]. At baseline, 30-, 60- and 84-months follow-up all study participants were asked knee-specific symptom questions. Participants with positive responses to having knee pain on most days in the past 30 days were classified as having current knee symptoms; in accordance with previous methods [24,25]. In addition to the clinical visit, participants also completed an assessment of frequent knee pain by telephone interview approximately 30-days prior to each clinic visit. A stricter definition of persistent, frequent knee pain was used and was defined as having positive responses to the NHANES questions at both the telephone and clinical visits [[26], [27], [28], [29]].

### 2.4 Exposures: ankle and foot symptoms

Our three exposures (participant-level) were baseline: i) ankle pain, ii) foot pain and iii) a composite of ankle and foot pain (i.e., on the same side or on opposing sides).

Side-specific ankle and foot symptoms were recorded at baseline. Participants who reported having at least a single joint with pain for most days in the previous month across the whole body were encouraged to complete a pain diagram. On the pain diagram, participants were encouraged to indicate in which joint(s) they experienced pain on most days during the past 30 days. The feet comprised forefoot and plantar foot regions and were assessed using a separate, foot-specific pain diagram. Participants who positively reported ankle pain on the pain diagram (either/both ankles) were defined as having current ankle pain whilst participants who positively indicated having foot pain (across any foot region) were classified as having current foot pain. Our third outcome was a composite of ankle and foot pain. Only participants with complete ankle and foot symptom data were included.

### 2.5 Outcome

We investigated the incidence of: i) RKOA, ii) symptomatic RKOA (both RKOA and knee pain in the same knee) and iii) frequent knee pain (only) in participants that had no evidence of RKOA and knee pain in both knees at baseline. Incidence was examined at participant level; i.e., it was not a requirement to have, for example, disease incidence on the painful joint (ankle/foot) side in order to be classified as an incident case.

i) Incident RKOA

Incident RKOA was defined as the occurrence of RKOA (KL ​≥ ​2) in either/both knee(s) during follow-up.

ii) Incident symptomatic RKOA

Incident symptomatic RKOA was defined as the occurrence of a combination of frequent knee symptoms and RKOA (KL score ≥2) in the same knee at one of more of the follow-up visits.

iii) Incident frequent knee pain

Incident frequent knee pain was defined as the occurrence of knee pain in either/both knees at one or more of the follow-up visits.

### 2.6 Statistical methods

Participant characteristics were summarized with normally distributed variables presented as means and standard deviations (SD), non-normally distributed variables presented as medians and interquartile range (IQR) and, categorical variables presented as frequencies and percentages. To examine the relationship between our three exposures (i.e. ankle, foot and, and ankle and foot pain) and incident outcomes, we performed logistic regression analyses. Results were presented as odds ratios (OR) with 95% confidence intervals (CIs) for crude and adjusted models.

We simultaneously controlled for baseline age (continuous), BMI (continuous), sex (categoric, 0 ​= ​female and 1 ​= ​male) and race (categoric, 0 ​= ​white/Caucasian, 1 ​= ​black/African American, 2 ​= ​other) in our models. In addition, comorbidities were assessed using a modified version of the Charlson comorbidity index (CCI) [30], and we dichotomised the sample (range: 0–8) into those with no comorbidities and those with ≥1 comorbidity. Further, we mutually adjusted for the presence of baseline foot pain (participant-level, yes/no) in analyses where ankle pain was our exposure and vice-versa.

For the symptomatic RKOA analysis, we imputed missing follow-up RKOA status in left/right knees at 30-, 60- and 84-months follow-up visits only where RKOA status was known either at the previous visit (i.e., RKOA positive) or at both adjacent visits (i.e., RKOA negative). For example, if the 60-month follow-up visit was missing but the study participant was positive for RKOA at the 30-month visit, then we would impute the 60-month visit. We included participants with missing RKOA or knee symptom data in cases where it was still possible to determine symptomatic RKOA status (e.g., RKOA negative, missing knee symptom data). In most cases, less than 5% of the eligible study sample were imputed.

We also conducted a sensitivity analyses in which we used a stricter definition of persistent, frequent knee pain (i.e., positive responses for knee pain at the telephone and clinical visit) for both incident symptomatic RKOA and incident knee pain outcomes.

## 3 Results

**Fig. 1:**
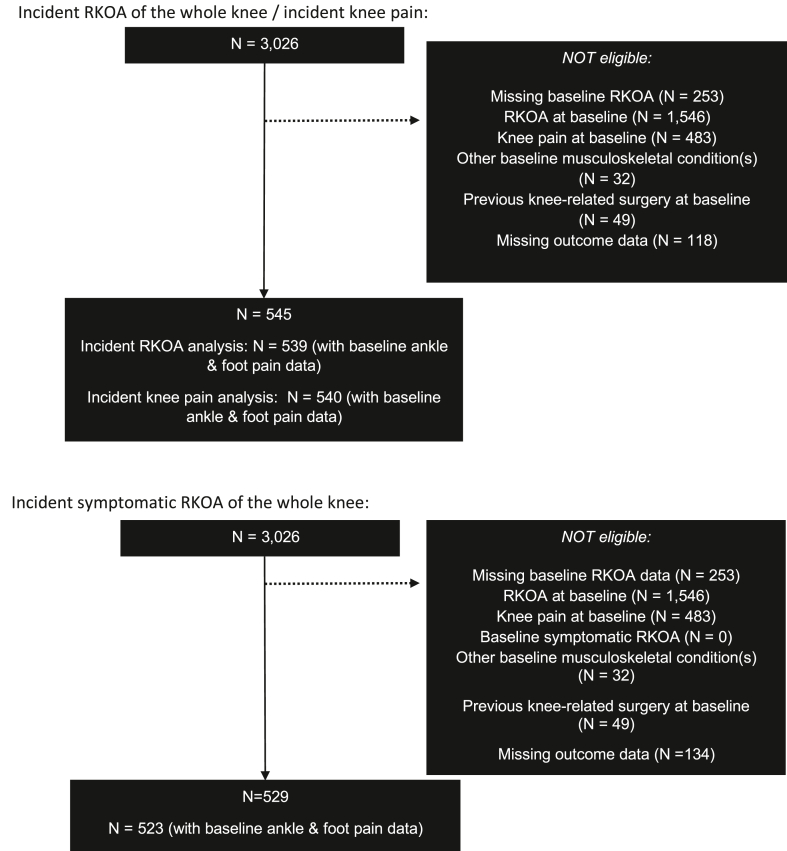
Flow chart of participants eligible for study investigation.

At baseline, 539 (17.8%), 523 (17.3%) and 540 (17.9%) participants were eligible for incident RKOA, symptomatic RKOA and frequent knee pain analyses, respectively. A flowchart of participants eligible for each analysis is shown in Fig. 1. Whilst participant eligibility criteria were the same for all analyses (i.e. no evidence of RKOA and knee pain in both knees at baseline), the number of participants that were included in the three main analyses varied due to the number of complete cases (e.g. those with outcome data).

Characteristics of eligible participants with ankle and foot pain data are shown in Table 1. In participants with ankle/foot pain data at baseline, incident RKOA, symptomatic RKOA and frequent knee pain occurred in 169 (31.0%), 66 (12.5%) and 249 (45.7%) participants during follow-up, respectively.

**Table 1:** Baseline demographics of eligible study participants.

Variable	MOST		
	No RKOA (N ​= ​545)	No Symptomatic RKOA (N ​= ​529)	No Knee Pain (N ​= ​545)
Age (years) (median, IQR)	60 (54–66)	60 (54–66)	60 (54–66)
Sex, n (% female)	312 (57.3)	303 (57.3)	312 (57.3)
BMI (kg/m^2^) (mean, SD)	28.9 (4.3)	28.8 (4.3)	28.8 (4.3)
**Charlson Comorbidity Score, n** (%)			
*0*	431 (79.1)	418 (79.0)	429 (78.7)
*≥1*	114 (20.9)	111 (21.0)	116 (21.3)
**Race**			
*White or Caucasian*	492 (90.3)	480 (90.7)	490 (89.9)
*Black or African American*	45 (8.3)	41 (7.8)	47 (8.6)
*Other*	8 (1.4)	8 (1.5)	8 (1.5)
**Ankle pain, n** (%)			
*Neither ankle*	464 (85.2)	453 (85.7)	462 (84.8)
*Right only*	17 (3.1)	16 (3.0)	17 (3.1)
*Left only*	11 (2.0)	10 (1.9)	11 (2.0)
*Both*	47 (8.6)	44 (8.3)	50 (9.2)
*Missing*	6 (1.1)	6 (1.1)	5 (0.9)
**Foot pain, n** (%)			
*Neither foot*	372 (68.3)	363 (68.6)	370 (67.9)
*Right only*	45 (8.3)	45 (8.5)	47 (8.6)
*Left only*	41 (7.5)	39 (7.4)	41 (7.5)
*Both*	81 (14.8)	76 (14.4)	82 (15.1)
*Missing*	6 (1.1)	6 (1.1)	5 (0.9)
**Foot and ankle pain, n** (%)			
*No*	362 (66.4)	353 (66.7)	358 (65.7)
*Yes*	65 (11.9)	60 (11.4)	66 (12.1)
*Other combination/Missing*	118 (21.7)	116 (21.9)	121 (22.2)

Abbreviations: BMI, body mass index; SD, standard deviation; IQR, inter-quartile range; RKOA, radiographic knee osteoarthritis (OA).

In multivariate analysis adjusting for baseline age, BMI, sex, race and Charlson comorbidity score, baseline ankle pain (OR: 1.11, 95% CI 0.65 to 1.88), foot pain (1.00, 95% CI 0.66 to 1.50) and, ankle and foot pain (1.01, 95% CI 0.57 to 1.82) were not statistically significantly associated with increased odds of incident RKOA; see Table 2. No statistically significant associations were observed between exposures and incident RKOA after further adjustment for the presence of ankle or foot pain.

**Table 2:** Odds ratio's for the association between baseline ankle, foot and ankle/foot pain and incident RKOA.

Exposure	Univariate	Multivariate^1^	Multivariate^2^
Ankle Pain	N ​= ​539	N ​= ​539	N ​= ​539
*No (n* ​= ​*464, 138)*	reference	reference	reference
*Yes (n* ​= ​*75, 29)*	1.49 (0.90–2.47), 0.12	1.11 (0.65–1.88), 0.71	1.15 (0.62–2.11), 0.66
Foot Pain	N ​= ​539	N ​= ​539	N ​= ​539
*No (n* ​= ​*372, 112)*	reference	reference	reference
*Yes (n* ​= ​*167, 55)*	1.14 (0.77–1.69), 0.51	1.00 (0.66–1.50), 0.98	0.95 (0.59–1.51), 0.81
Ankle and Foot Pain	N ​= ​427	N ​= ​427	–
*No (n* ​= ​*362, 107)*	reference	reference	–
*Yes (n* ​= ​*65, 24)*	1.40 (0.80–2.42), 0.24	1.01 (0.57–1.82), 0.96	–

All results presented as odds ratios (OR) with 95% confidence intervals and *P*-values.

*N*-values are presented as the number of participants for the given category with the number of incident cases.

Abbreviations: RKOA, radiographic knee osteoarthritis; BMI, body mass index.

^1^Adjusted for age, sex, BMI, race and Charlson Comorbidity score (dichotomised).

^2^Adjusted for sex, age, BMI, race, Charlson Comorbidity score (dichotomised) and were mutually adjusted for the other type of joint pain (ankle or foot).

In multivariate analysis, baseline ankle pain (OR: 2.53, 95% CI 1.34 to 4.80) and, ankle and foot pain (2.30, 95% CI 1.13 to 4.66) were statistically significantly associated with >2-fold increased odds of incident symptomatic RKOA, though no statistically significant association was observed for foot pain (1.22, 95% CI 0.70 to 2.13); see Table 3. After further adjustment for the presence of foot pain, the relationship between ankle pain and incident symptomatic RKOA remained (3.06, 95% CI 1.40 to 6.68). In a sensitivity analysis using a stricter definition of frequent knee pain, baseline ankle pain and, ankle and foot were statistically significantly associated with incident symptomatic RKOA; see Supplementary Table 1. No statistically significant association was observed for foot pain.

**Table 3:** Odds ratio's for the association between baseline ankle, foot and ankle/foot pain and incident symptomatic RKOA.

Exposure	Univariate	Multivariate^1^	Multivariate^2^
Ankle Pain	N ​= ​523	N ​= ​523	N ​= ​523
*No (n* ​= ​*453, 47)*	reference	reference	reference
*Yes (n* ​= ​*70, 18)*	**2.99 (1.62 to 5.53), 0.001**	**2.53 (1.34 to 4.80), 0.004**	**3.06 (1.40 to 6.68), 0.005**
Foot Pain	N ​= ​523	N ​= ​523	N ​= ​523
*No (n* ​= ​*363, 41)*	reference	reference	reference
*Yes (n* ​= ​*160, 24)*	1.39 (0.81–2.38), 0.24	1.22 (0.70–2.13), 0.48	0.75 (0.37–1.50), 0.41
Ankle and Foot Pain	N ​= ​413	N ​= ​413	–
*No (n* ​= ​*353, 38)*	reference	reference	–
*Yes (n* ​= ​*60, 15)*	**2.76 (1.41 to 5.42), 0.003**	**2.30 (1.13 to 4.66), 0.02**	–

All results presented as odds ratios with 95% confidence intervals and *P*-values.

*N*-values are presented as the number of participants for the given category with the number of incident cases.

Statistically significant results, at the ≥0.05 level, are shown in bold.

Abbreviations: RKOA, radiographic knee osteoarthritis; BMI, body mass index.

^1^Adjusted for age, sex, BMI, race and Charlson Comorbidity score (dichotomised).

^2^Adjusted for sex, age, BMI, race, Charlson Comorbidity score (dichotomised) and were mutually adjusted for the other type of joint pain (ankle or foot).

In multivariate analysis, baseline ankle pain (4.70, 95% CI 2.65 to 8.33), foot pain (1.92, 95% CI 1.32 to 2.79) and, ankle and foot pain (4.56, 95% 2.45 to 8.50) were statistically significantly associated with incident knee pain, respectively. See Table 4. However, after further adjustment for ankle or foot pain, statistical significance for the relationship between foot pain and incident knee pain was lost and the effect was reduced (1.24, 95% CI 0.81 to 1.90) though remained for ankle pain and frequent knee pain. These data suggest that the effect of foot pain on incident knee pain can be explained largely by the presence of current ankle pain. In our sensitivity analysis using a stricter definition of frequent knee pain, ankle pain and, ankle and foot pain were statistically significantly associated with incident knee pain, respectively. No association was observed for foot pain.

**Table 4:** Odds ratio's for the association between baseline ankle, foot and ankle/foot pain and incident knee pain.

Exposure	Univariate	Multivariate^1^	Multivariate^2^
Ankle Pain	N ​= ​540	N ​= ​540	N ​= ​540
*No (n* ​= ​*462, 188)*	reference	reference	reference
*Yes (n* ​= ​*78, 60)*	**4.86 (2.78 to 8.49), 0.001**	**4.70 (2.65 to 8.33), 0.001**	**4.13 (2.21 to 7.72), 0.001**
Foot Pain	N ​= ​540	N ​= ​540	N ​= ​540
*No (n* ​= ​*370, 149)*	reference	reference	reference
*Yes (n* ​= ​*170, 99)*	**2.07 (1.43 to 2.99), 0.001**	**1.92 (1.32 to 2.79), 0.001**	1.24 (0.81–1.90), 0.32
Ankle and Foot Pain	N ​= ​424	N ​= ​424	–
*No (n* ​= ​*358, 139)*	reference	reference	–
*Yes (n* ​= ​*66, 50)*	**4.92 (2.70 to 8.99), 0.001**	**4.56 (2.45 to 8.50), 0.001**	–

All results presented as odds ratios with 95% confidence intervals and *P*-values.

*N*-values are presented as the number of participants for the given category with the number of incident cases.

Statistically significant results, at the ≥0.05 level, are shown in bold.

Abbreviations: RKOA, radiographic knee osteoarthritis; BMI, body mass index.

^1^Adjusted for age, sex, BMI, race and Charlson Comorbidity score (dichotomised).

^2^Adjusted for sex, age, BMI, race, Charlson Comorbidity score (dichotomised) and were mutually adjusted for the other type of joint pain (ankle or foot).

## 4 Discussion

We examined the relationship between baseline ankle pain, foot pain and ankle/foot symptoms and, incident knee outcomes in participants with no evidence of RKOA and knee symptoms, respectively. We observed statistically significant, and clinically meaningful, relationships between baseline ankle pain and, ankle pain with foot pain and incident symptomatic RKOA and knee pain. No statistically significant associations were observed between any of our exposures and incident RKOA and, we found no statistically significant associations for foot pain after adjustment for participant characteristics and ankle pain.

Previous studies suggest that ankle/foot symptoms in either or both feet may be associated with knee OA development [13] and, worsening of knee pain and radiographic change in patients with symptomatic knee OA [14]. Paterson et al. reported that in participants without frequent knee symptoms and evidence of RKOA, ankle and/or foot pain was associated with an increased risk of incident knee symptoms (1.55, 95% CI 1.10 to 2.19) and incident symptomatic RKOA (3.28, 95% CI 1.69 to 6.37) [13]. We observed similar effects for baseline ankle symptoms though the size and magnitude of the effects were considerably greater for incident knee pain (4.13, 95% CI 2.21 to 7.72) and incident symptomatic RKOA (3.06, 95% CI 1.40 to 6.68). The likely explanation for these findings is that whilst our eligible study population was smaller (N ​= ​545/529 vs. N ​= ​1020), the prevalence of ankle/foot symptoms was comparable to that reported by Paterson *et al* [13]. The benefit to our study was that we were able to examine the individual effects of baseline ankle and foot pain on incident outcomes. In agreement with findings reported by Paterson *et al* [13,14], we observed a relationship between ankle and foot symptoms and incident symptomatic RKOA and knee pain. Interestingly, the magnitude of the effect for the association between ankle pain and, ankle pain with foot pain (person-level) and incident symptomatic RKOA and incident frequent knee pain were similar. Our data would suggest that the relationship with incident symptomatic RKOA and knee pain is driven mostly by ankle pain.

Fluctuations in joint pain in patients with knee OA are common [31] but risk factors for pain fluctuation are poorly understood. Subsequently, to best examine the relationship between ankle pain with/without foot pain and incident knee outcomes, we used two measures of frequent knee symptoms. These included reporting knee pain at a single, clinical visit which is an accepted approach for assessing symptoms in OA [32] and, a stricter definition of knee pain (positive responses at both telephone and clinic visit). After adjustment for patient demographics and foot pain, baseline ankle pain was statistically significantly associated with incident knee pain when using both the standard definition (4.13, 95% CI 2.21 to 7.72) and stricter definition (2.77, 95% CI 1.52 to 5.06) of knee pain. Both definitions of knee pain may capture different pain phenotypes however, the consistency in the direction of the effect and, to some extent the magnitude of the effect, gives confidence in the observed relationship between ankle pain and incident knee pain. Whilst we did not observe a relationship between exposures and incident RKOA, we did observe a statistically significant relationship between baseline ankle pain and, ankle and foot pain and incident symptomatic RKOA; this group includes the development of RKOA with incident knee symptoms. Whilst these data suggest that baseline ankle pain may not be associated with incident RKOA, they further support a relationship between ankle pain and incident knee pain.

There are several strengths to this study. First, to our knowledge, this is the first study to examine the individual effects of baseline ankle, foot and, ankle and foot pain on incident knee outcomes using data from a large, well-characterised long-term study of knee OA. Further, we were able to make full use of the repeated follow-up visits and subsequently, we were able to identify cases of fluctuating knee pain (i.e. incident symptomatic RKOA/incident knee pain cases where painful symptoms were present at one of the follow-up visits).

There are several potential limitations to our study. First, ankle and foot pain were assessed using a pain diagram; specifically, ankle pain status was examined using a whole-person pain diagram whilst foot pain was assessed using a separate, foot-specific pain diagram. Unlike the knees and hips, localizing painful sites across the feet and ankles can be difficult with differentiation of ‘ankle’ pain from ‘foot’ pain often challenging (e.g. subtalar joint pain from midfoot pain). There is evidence to suggest that the prevalence of symptom-related outcomes or exposures is influenced by the method of data capture, i.e., a pain diagram versus written questions [33], with the most accurate form of assessment thought to be a written question. In order to assess the accuracy of the pain diagram, we compared the prevalence of current pain for a joint in which we had both separate written pain questions and pain status determined by diagram; i.e., the knee. Using separate written symptom questions as the reference, current pain status for the knee joint at a person-level was correctly identified as ‘true positive’ or ‘true negative’ in 395 (73.3%) participants eligible for incident RKOA analysis. Based on these data, we can expect that our estimates of baseline ankle and/or foot pain were underestimated however, most cases were correctly identified by pain diagram. Lastly, our findings for incident knee pain were limited to participants without RKOA at baseline and for incident RKOA, were limited to participants without frequent pain. Therefore, our findings may be generalizable to only a subpopulation of people who develop these outcomes. More so, whilst our study sample were free of RKOA and knee pain at baseline, they were an at-risk population (e.g. high BMI) and therefore, were not reflective of a healthy population. Furthermore, several of our analyses had few incident cases, particularly those for incident symptomatic RKOA. This is reflected in wide confidence intervals particularly for the ankle/foot pain and incident knee pain analyses. Although the confidence intervals were wide, the direction and magnitude of these effects were similar across multivariate and sensitivity analyses giving confidence to our findings.

The individual contributions of the ‘rearfoot’, including the talus and calcaneus bones and the subtalar and talocrural (ankle) joints, to the development of knee OA are not well understood. The main reasons for this being that most epidemiological studies assess pain of the ‘ankle’ and/or ‘feet’. However, specific components of the ‘rearfoot’ including subtalar and transverse tarsal joints have been shown to influence lower-limb alignment and knee loading. For example, the importance of the subtalar joint on gait can be seen most effectively in those who have undergone ankle and subtalar joint replacement [34]. Future epidemiological studies should aim to include individual assessments of the joints that comprise the ankle.

## 5 Conclusion

Our findings support a relationship between baseline ankle pain and incident symptomatic RKOA and knee pain, respectively. There were no statistically significant associations between ankle, foot and, ankle and foot pain and incident RKOA. Future observational studies should include ankle joint-specific symptom questions to advance understanding of the biomechanical pathophysiology of knee OA.

## Author contributions

Conception and Design: TAP, NAS, MCN, LG. Analysis and interpretation of data: TAP and MCN. Drafting Article: TAP. Critical revision of article: all authors. Final Approval: all authors.

## Role of funding source

This study was supported financially by the Centre for Sport, Exercise and Osteoarthritis Research 10.13039/501100012041Versus Arthritis (Grant reference 21595). The funders were not involved in the study design, data collection and interpretation of study results.

## Declaration of competing interest

TAP, NAS, CB, LG and MCN declare no conflicts of interest. NA has received honorariums from Novartis, Alliance for Better Health, and Lilly; held advisory board positions (which involved receipt of fees) at Merck, Merck Sharp and Dohme, Roche, Novartis, Smith and Nephew, Q-MED, Nicox, Servier, GlaxoSmithKline, Schering-Plough, Pfizer, and Rottapharm; and received consortium research grants from Alliance for Better Bone Health, Amgen, Novartis, Merck Sharp and Dohme, Servier, Eli Lilly, and GlaxoSmithKline; he has no other relationships or activities that could appear to have influenced the submitted work.

## Availability of data and materials

All data generated and analysed in this study are available upon reasonable request. Access to data generated in this report should be sent to the corresponding author at thomas.perry@ndorms.ox.ac.uk whilst requests for MOST data should be submitted to the cohort principle investigators.

## Public and patient involvement (PPI) statement

PPI was not required nor involved with any aspect of the work presented.
