# Supplementary material for: Foot and ankle pain and risk of incident knee osteoarthritis and knee pain: Data from the Multicentre Osteoarthritis Study
Source: Osteoarthr Cartil Open. 2021 Aug 27;3(4):100210. doi: 10.1016/j.ocarto.2021.100210 (PMC8683744; doi:10.1016/j.ocarto.2021.100210)
Supplement: Multimedia component 1 [file mmc1.docx]

| Supplementary Table 1: *Association between current ankle, foot and ankle/foot pain and incident symptomatic RKOA: using a stricter definition of knee pain.* | | | | | |
| --- | --- | --- | --- | --- | --- |
| Exposure | **Univariate** | **Multivariate^1^** | | **Multivariate^2^** | |
| Ankle Pain | N = 525 | N = 525 | | N = 525 | |
| *No (n = 455, 34)* | reference | reference | | reference | |
| *Yes (n = 70, 14)* | **3.10 (1.57 to 6.12), 0.001** | **2.67 (1.31 to 5.43), 0.007** | | **3.02 (1.26 to 7.23), 0.01** | |
| Foot Pain | **N = 525** | **N = 525** | | **N = 525** | |
| *No (n = 363, 29)* | reference | reference | | reference | |
| *Yes (n = 162, 19)* | 1.53 (0.83 to 2.82), 0.17 | 1.34 (0.72 to 2.52), 0.36 | | 0.82 (0.38 to 1.80), 0.62 | |
| Ankle and Foot Pain | N = 413 | N = 413 | | - | |
| *No (n = 353, 27)* | reference | | reference | | - |
| *Yes (n = 60, 12)* | **3.02 (1.43 to 6.36), 0.004** | | **2.56 (1.17 to 5.62), 0.02** | | - |
| All results presented as odds ratios with 95% confidence intervals and P-values.  N-values are presented as the number of participants for the given category with the number of incident cases.  Statistically significant results, at the ≥0.05 level, are shown in bold.  Abbreviations: RKOA, radiographic knee osteoarthritis; BMI, body mass index.  ^1^Adjusted for age, sex, BMI, race and Charlson Comorbidity score (dichotomised).  ^2^Adjusted for sex, age, BMI, race, Charlson Comorbidity score (dichotomised) and were mutually adjusted for the other type of joint pain. | | | | | |
