# Supplementary material for: Foot and ankle pain and risk of incident knee osteoarthritis and knee pain: Data from the Multicentre Osteoarthritis Study
Source: Osteoarthr Cartil Open. 2021 Aug 27;3(4):100210. doi: 10.1016/j.ocarto.2021.100210 (PMC8683744; doi:10.1016/j.ocarto.2021.100210)
Supplement: Multimedia component 2 [file mmc2.docx]

| Supplementary Table 2: *Association between current ankle, foot and ankle/foot pain and incident knee pain; using a stricter definition of knee pain.* | | | | | |
| --- | --- | --- | --- | --- | --- |
| Exposure | **Univariate** | **Multivariate^1^** | | **Multivariate^2^** | |
| Ankle Pain | N = 519 | N = 519 | | N = 519 | |
| *No (n = 447, 119)* | reference | reference | | reference | |
| *Yes (n = 72, 41)* | **3.65 (2.19 to 6.08), 0.001** | **3.29 (1.93 to 5.59), 0.001** | | **2.77 (1.52 to 5.06), 0.001** | |
| Foot Pain | N = 519 | N = 519 | | N = 519 | |
| *No (n = 357, 92)* | reference | reference | | reference | |
| *Yes (n = 162, 68)* | **2.08 (1.41 to 3.08), 0.001** | **1.89 (1.26 to 2.82), 0.002** | | 1.32 (0.83 to 2.10), 0.24 | |
| Ankle and Foot Pain | N = 409 | N = 409 | | - | |
| *No (n = 347, 85)* | reference | | reference | | - |
| *Yes (n = 62, 34)* | **3.74 (2.15 to 6.53), 0.001** | | **3.19 (1.78 to 5.74), 0.001** | | - |
| All results presented as odds ratios with 95% confidence intervals and P-values.  N-values are presented as the number of participants for the given category with the number of incident cases.  Statistically significant results, at the ≥0.05 level, are shown in bold.  Abbreviations: RKOA, radiographic knee osteoarthritis; BMI, body mass index.  ^1^Adjusted for age, sex, BMI, race and Charlson Comorbidity score (dichotomised).  ^2^Adjusted for sex, age, BMI, race, Charlson Comorbidity score (dichotomised) and were mutually adjusted for the other type of joint pain. | | | | | |
